# Post-exposure treatment of Ebola virus disease in guinea pigs using EBOTAb, an ovine antibody-based therapeutic

**DOI:** 10.1038/srep30497

**Published:** 2016-07-28

**Authors:** Stuart D. Dowall, Andrew Bosworth, Emma Rayner, Irene Taylor, John Landon, Ian Cameron, Ruth Coxon, Ibrahim Al Abdulla, Victoria A. Graham, Graham Hall, Gary Kobinger, Roger Hewson, Miles W. Carroll

**Affiliations:** 1 Public Health England, Porton Down, Salisbury, Wiltshire, SP4 0JG, UK; 2 MicroPharm Ltd, Station Road, Newcastle Emlyn, Dyfed, SA38 9BY, UK; 3 Public Health Agency of Canada, 1015 Arlington Street, Winnipeg, Manitoba R3E 3R2, Canada; 4 University of Manitoba, 745 Bannatyne Avenue, Winnipeg, Manitoba R3E 0J9, Canada

## Abstract

Ebola virus (EBOV) is highly pathogenic, with a predisposition to cause outbreaks in human populations accompanied by significant mortality. An ovine polyclonal antibody therapy has been developed against EBOV, named EBOTAb. When tested in the stringent guinea pig model of EBOV disease, EBOTAb has been shown to confer protection at levels of 83.3%, 50% and 33.3% when treatment was first started on days 3, 4 and 5 post-challenge, respectively. These timepoints of when EBOTAb treatment was initiated correspond to when levels of EBOV are detectable in the circulation and thus mimic when treatment would likely be initiated in human infection. The effects of EBOTAb were compared with those of a monoclonal antibody cocktail, ZMapp, when delivered on day 3 post-challenge. Results showed ZMapp to confer complete protection against lethal EBOV challenge in the guinea pig model at this timepoint. The data reported demonstrate that EBOTAb is an effective treatment against EBOV disease, even when delivered late after infection.

Ebola virus (EBOV) has recently been responsible for its largest outbreak in West Africa, first recognised in March 2014^1^, causing more deaths than all previously known outbreaks combined. Whilst the first outbreak of EBOV was identified in 1976^2^, there are still no approved therapeutics; however, during the 2014 EBOV outbreak the World Health Organisation approved immunotherapy in the form of homologous polyclonal antibodies (pAb)^3^. Nevertheless, due to difficulties with the human-derived antibody treatments^4^, alternative immunotherapeutic strategies are required.

In order to be of value in EBOV infection, treatments should be tested after exposure to the virus in order to demonstrate therapeutic effects. Post-exposure therapies against filoviruses described in human clinical trials and/or animal model systems have previously been reviewed^5^ and consist of: recombinant proteins involved with anticoagulation^6^ and human activated protein C^7^; RNA interference by phosphorodiamidate morpholino oligomers^8^ and stable nucleic acid-lipid particles targeting the EBOV L protein^9^,^10^; mannose-binding lectin^11^; and small molecule inhibitors^12^,^13^. These treatments range from treating clinical symptoms, inhibiting viral processes, boosting host immune responses and limiting viremia^14^. Vaccination approaches have also been demonstrated to confer post-exposure protection against EBOV, such as a recombinant vesicular stomatitis virus vector expressing the EBOV glycoprotein which protected 50% of guinea pigs following treatment up to 24 hours after lethal challenge^15^.

Cocktails of monoclonal antibodies (mAb) are currently the most studied post-exposure EBOV treatments reported and are the only therapy that has demonstrated substantial benefits in non-human primates when administered greater than 24 hours post-EBOV exposure^16^. Verified protection as late as 3 days post-infection has been reported in the guinea pig model^17^. Based on success in non-human primates, ZMapp and ZMAb have been used under emergency compassionate protocols in humans to treat EBOV infections originating from outbreak (25 were treated on compassionate ground, 22 survived and only 1 died after receiving at least 2 doses), six patients have been treated with ZMAb, with all surviving and all administrations were reported as well tolerated^18^. While still not clear if the survival can be directly attributed to treatment with the mAb cocktails, this production and clinical testing of anti-EBOV cocktails is being accelerated. However, mAb therapies suffer from several disadvantages including high production costs and risk of escape mutants, particularly for RNA viruses such as EBOV which have high mutation frequencies^19^; polyclonal antibody (pAb) approaches are therefore an alternative option. An ovine pAb-based product, EBOTAb, has previously been described based on purified IgG from sheep immunised with mammalian-expressed recombinant EBOV glycoprotein^20^. This approach offers a cost-effective method of treating EBOV infection and is economically viable for developing regions facing epidemic EBOV disease. Similar intact ovine pAb have been used in West Africa for several years to treat >40,000 patients envenomated by carpet vipers, with the resultant product EchiTAb being one of the most cost-effective therapies currently available^21^.

EBOTAb has previously been demonstrate to bind to both the GP_1_ and GP_2_ subunits of the EBOV glycoprotein^20^; and since this is a pAb preparation it contains antibodies against multiple epitopes. This reduces the likelihood that escape mutations of EBOV can arise as has been reported for individual mAb contained within ZMapp^22^. Since different epitopes are recognised at different stages of viral infection, the pAb approach is likely to confer multiple effects including inhibition of host cell attachment, obstructing enzymatic cleavage and blocking the cleaved forms of glycoprotein, thus obstructing the activation of endosomal virus-cell fusion while limiting the emergence of escape mutants^23^,^24^.

Whilst an initial study documented the use of EBOTAb delivered 6, 48 and 72 hours post-challenge, this report covers our assessment of the protective efficacy of EBOTAb up to 5 days post-challenge and compares EBOTAb delivery with ZMapp, a mAb-based therapy.

## Results

### Efficacy of antibody treatment delivered 3 days post-EBOV challenge

**Figure 1:**
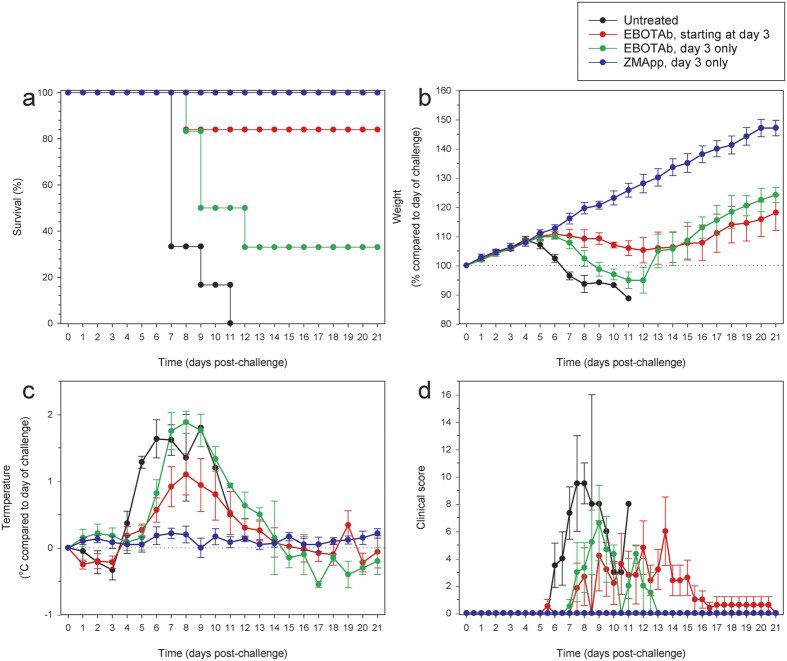
Survival and clinical observations of EBOV-challenged guinea pigs after treatment with EBOTAb or ZMapp starting after 3 days. Guinea pigs were challenged with EBOV and treated with EBOTAb or ZMapp after 3 days. One group received 0.5 ml (51 mg/ml, equating to 75–86 mg/kg) EBOTAb twice per day on days 3, 4, 5, 7, 9 and 10 post-challenge. Two groups received 0.5 ml of EBOTAb (48 mg/ml, equating to 68–77 mg/kg) and ZMapp (3.4 mg/ml, equating to 5–6 mg/kg), respectively, in each of three doses delivered within 10 hours with the concentrations arranged so the same amount of EBOV-specific antibody was present and both groups received 10 mg of specific-antibody on day 3 post-challenge. (**a**) Survival analysis between EBOTAb and ZMapp treated groups compared to untreated animals (n = 6 per group). (**b**) Weight changes, showing percentage differences from values on the day of challenge. (**c**) Temperature differences in animals compared to values on the day of challenge. (**d**) Clinical scores of animals after challenge. In panels **b**–**d**, mean results are shown for animals still surviving in all groups, with error bars denoting standard error.

The EBOTAb or ZMapp preparation was first delivered to guinea pigs on the third day after infection with a lethal dose of EBOV. Survival analysis showed that all untreated animals met humane endpoints by day 11 (Fig. 1a). Treatment with EBOTAb starting on day 3 and with repeat administrations on days 4, 5, 7, 9 and 11 demonstrated 83.3% survival (Fig. 1a), a significance different as compared to untreated animals (P = 0.003, Log-Rank survival analysis). When EBOTAb was given on day 3 post-challenge alone, survival was reduced to 33% but this was still significant compared to the untreated controls (P = 0.032, Log-Rank survival analysis). ZMapp administered on day 3 resulted in complete protection (P < 0.001 compared to untreated animals, Log-Rank survival analysis). There was no statistical difference between the ZMapp-treated animals and those given EBOTAb at day 3 followed by repeat doses (P = 0.317, Log-Rank survival analysis). All animals treated with single or multiple doses of EBOTAb showed signs of disease, including weight loss and temperature increases (Fig. 1b,c, respectively). In the EBOTAb-treated groups there was an apparent delay in temperature spiking and a reduction in peak temperature in the group which received multiple doses of EBOTAb. Clinical signs were assessed in surviving animals from each of the groups and signs of illness were detected in the animals treated with EBOTAb, albeit at lower levels to untreated animals (Fig. 1d). In our analysis, ZMapp treated animals remained clinically healthy throughout the course of the study.

### Efficacy of EBOTAb treatment delivered 4 and 5 days post-EBOV challenge

**Figure 2:**
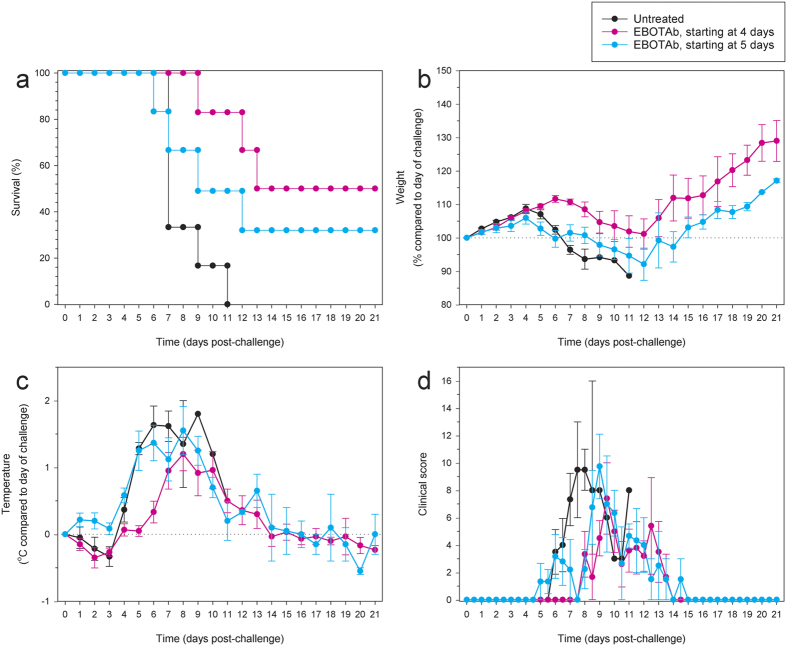
Survival and clinical observations of EBOV-challenged guinea pigs after treatment with EBOTAb starting after 4 and 5 days. Guinea pigs were challenged with EBOV and treated with EBOTAb starting after 4 or 5 days. 0.5 ml (51 mg/ml, equating to 71–84 mg/kg) EBOTAb was administered twice per day on three consecutive days followed a further 3 doses every other day. (**a**) Survival analysis between EBOTAb treated groups compared to untreated animals (n = 6 per group). (**b**) Weight changes, showing percentage differences from values on the day of challenge. (**c**) Temperature differences in animals compared to values on the day of challenge. (**d**) Clinical scores of animals after challenge. In panels B–D, mean results are shown for animals still surviving in all groups, with error bars denoting standard error.

EBOTAb was first delivered to guinea pigs 4 and 5 days after infection with a lethal dose of EBOV, with repeat (two per day) doses being given on three consecutive days followed by another six doses (two per day) every other day. Unfortunately, ZMapp was not available for this second experiment. Survival analysis showed that treatment with EBOTAb starting on day 4 and 5 resulted in 50% and 33% protection, respectively (Fig. 2a) (P = 0.002 and P = 0.134 compared to untreated animals, respectively; Log-Rank survival analysis). The survival in the EBOTAb treatment group starting on day 5 post-challenge was not significant due to a single guinea pig meeting humane clinical endpoint before any of the untreated control animals. All guinea pigs treated with EBOTAb showed signs of disease, including weight loss and temperature increases (Fig. 2b,c, respectively). Clinical signs were detected in the animals treated with EBOTAb at these later timepoints (Fig. 2d).

### Presence of EBOV RNA in the peripheral circulation

**Figure 3:**
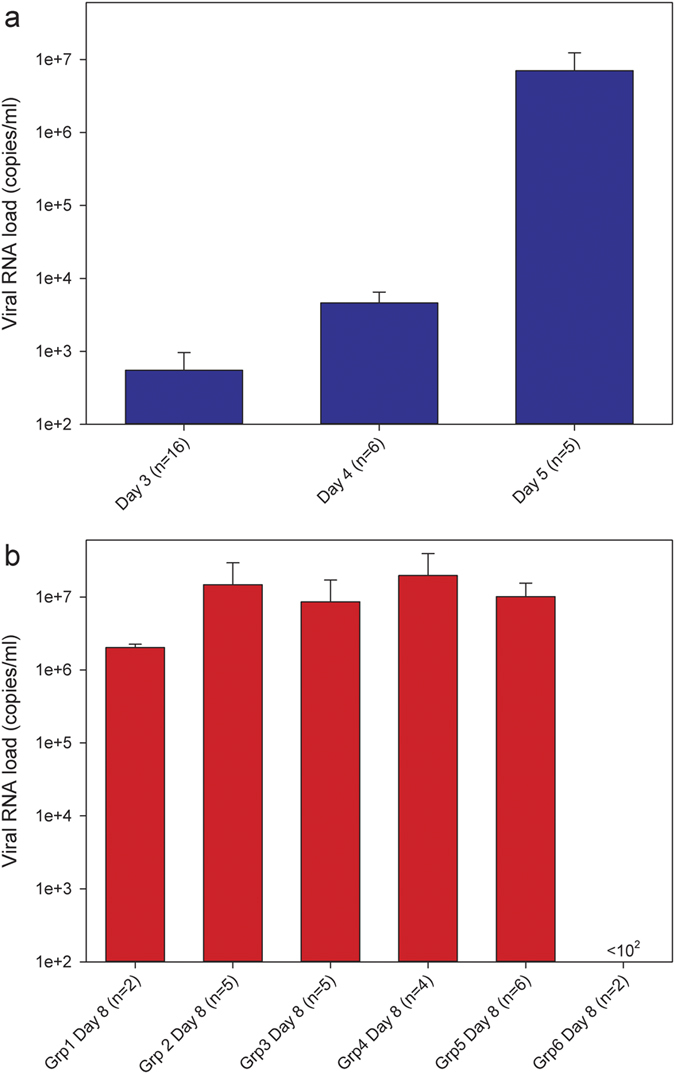
Viral genome copies in the blood of EBOV-challenged guinea pigs prior to treatment and on day 8 post-challenge. (**a**) RNA levels in the blood of EBOV challenged groups prior to administration of any antibody treatments on day 3, 4 and 5 post-challenge. (**b**) RNA levels in the blood at day 8 post-EBOV challenge of animals treated with EBOTAb and ZMapp. Bars show mean results with error bars denoting standard error.

Prior to the administration of antibody treatment on days 3–5 post-challenge, blood was withdrawn and the presence of EBOV RNA detected using a quantitative RT-PCR assay. Results showed viremia at day 3 post-challenge, which increased on day 4 and 5 post-challenge (Fig. 3a). On day 8 post-challenge, blood was sampled from all surviving animals with patent catheters and viral RNA levels assessed (Fig. 3b). The levels of viral RNA in EBOTAb treated animals were similar to untreated animals across all groups (P > 0.05, Mann-Whitney statistical test). No viral RNA was detectable within the limitations of the assay in the circulation of ZMapp treated animals 8 days post-challenge. Blood samples were collected at day 21 from the animals surviving until the scheduled end of the study and with their intravenous catheters still patent to allow blood withdrawal (EBOTAb given at days 3, 4 and 5 post-challenge, n = 3, n = 2 and n = 2, respectively; EBOTAb given on day 3 only, n = 1; and ZMapp given on day 3 only, n = 2). No viral RNA was detected in any of the blood of samples collected at 21 days post-challenge.

### Histological changes and presence of viral antigen in local sites of infection

**Figure 4:**
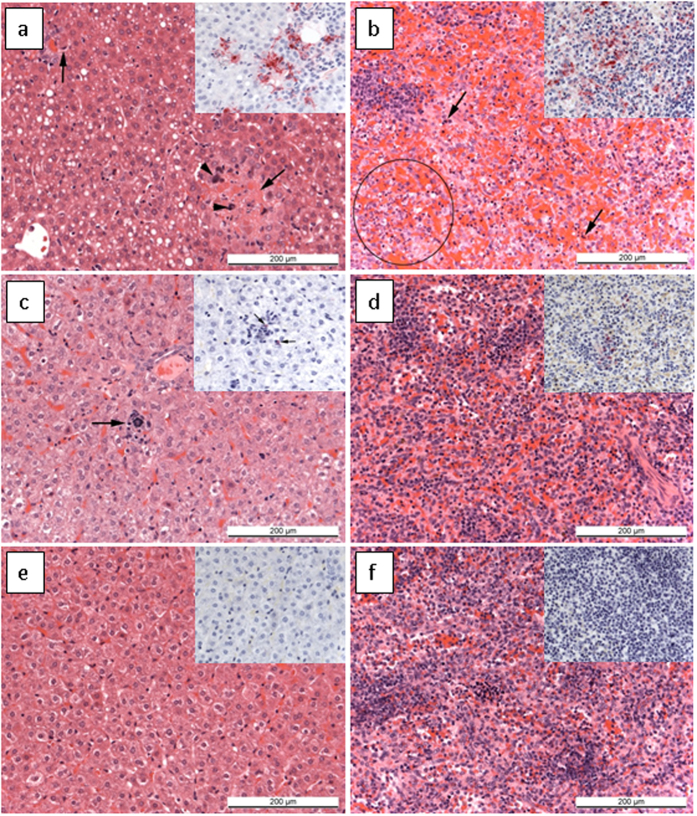
Histology and immunohistochemical staining of liver and spleen samples from EBOV infected guinea pigs treated with EBOTAb and ZMapp. (**a**) Liver, untreated (ID03904). Focal necrotic lesions (arrows) with mineralisation (arrowheads) and scattered, macrovesicular vacuolation of hepatocytes, consisent with fat. Inset, numerous cells staining strongly for Ebola viral antigen. (**b**) Spleen, untreated (ID03917). Marked congestion and single cell death within the red pulp (arrows), and patchy, PMN cell infiltrates (circle). Inset,numerous cells staining strongly for Ebola viral antigen. (**c**) Liver, EBOTAb day 3 treated (ID89303). Focal, mineralised areas (arrows). Inset, rare cells staining positive for Ebola viral antigen (arrows). (**d**) Spleen, EBOTAb day 3 treated (ID89258). Red pulp appears normal, with a minimal reduction in lymphocytes within the white pulp (not shown in image). Inset, small numbers of cells staining positive for Ebola viral antigen. (**e**) Liver, ZMapp treated on day 3 (ID03905). Normal. Inset, negative staining for viral antigen. (**f**) Spleen, ZMapp treated on day 3 (ID03905). Normal. Inset, negative staining for viral antigen. Images show H&E with insets of IHC stained samples.

Sections of liver and spleen were taken to assess the architecture of cells within the primary sites of EBOV infection in guinea pigs and stained for the presence of viral antigen (Table 1). In the untreated group, microscopic lesions and the presence of viral antigen were noted in the spleen and liver in all animals (Fig. 4a,b). In animals which received EBOTAb at day 3 post-challenge with repeat dosing, the treatment was generally effective (Fig. 4c,d); although one animal was severely affected (89314). Giving only a single dose of EBOTAb on day 3 or delaying dosing to days 4 and 5 resulted in microscopic lesions and EBOV antigen being detectable in most animals. Treatment with ZMapp showed fewer histological changes, although microscopic changes were noted, and no viral antigen was observed with immunohistochemical staining (Fig. 4e,f).

### Assessment of EBOV RNA levels in the liver and spleen of animals surviving at day 21 post-challenge

**Figure 5:**
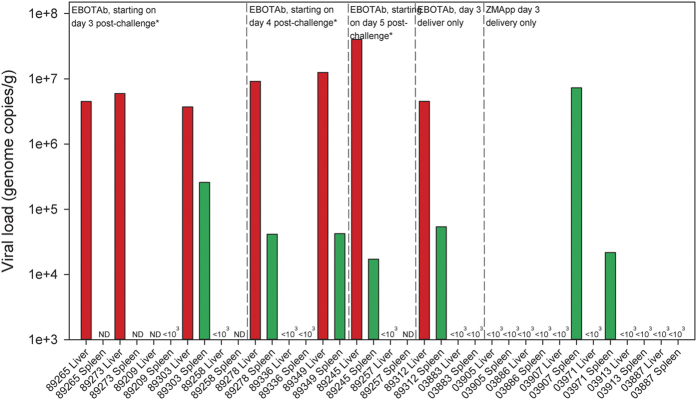
Viral genome copies in the liver and spleen of EBOV-challenged guinea pigs treated with EBOTAb and ZMapp and surviving to day 21. At the end of the scheduled study, sections of spleen and liver were taken from surviving animals and homogenised prior to assessment of the viral RNA levels by RT-PCR assay. Bars denote the mean value from triplicate wells quantified by RT-PCR. Abbreviations: ND, not done. *repeat dosing of EBOTAb was undertaken on 5 subsequent days.

At the scheduled end of the study, 21 days post-EBOV challenge, samples of liver and spleen were assessed for the presence of EBOV RNA in animals which survived. Several animals in the groups treated with EBOTAb had no evidence of viral RNA in the liver or spleen; however, in other animals viral RNA was detectable with higher levels being observed in the liver (Fig. 5). In the ZMapp treated group, EBOV RNA was detected in two animals, but only in the spleen.

### Determination of anti-EBOTAb specific antibodies

**Figure 6:**
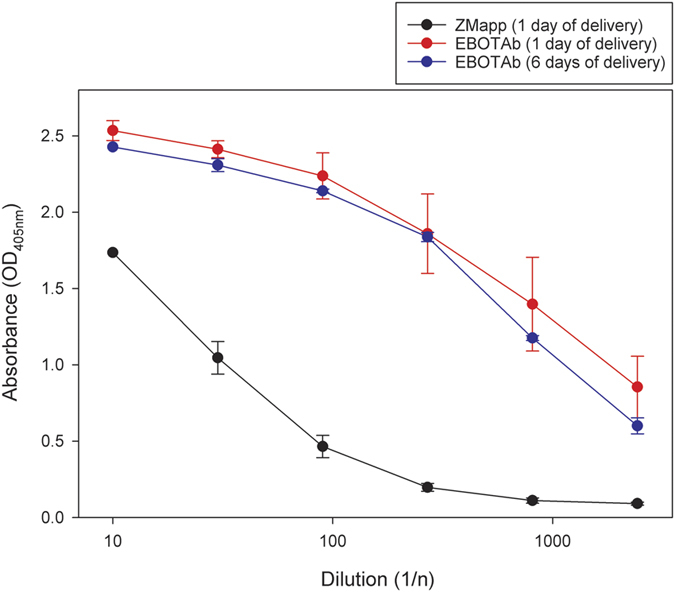
Measurement of anti-EBOTAb specific antibodies in the sera of animals surviving to day 21 post-challenge. At the end of the scheduled study, sera was isolated from animals where catheters retained patency from animals which had received ZMapp on a single day (n = 2), EBOTAb on a single (n = 2) or EBOTAb on six separate days (n = 8). Results show the mean values with error bars denoting standard error.

In animals which survived until day 21, blood was withdrawn from catheters that remained patent; 2 animals treated with ZMapp on one day, 2 animals treated with EBOTAb on one day and 8 animals which received EBOTAb on six separate days. EBOTAb-specific IgG was detected in the sera of animals treated with EBOTAb compared to those animals which received ZMapp as a control (Fig. 6). No difference in the generation of anti-EBOTAb antibody levels were observed in the groups treated on either a single day or those which received sequential treatments on 6 separate days.

## Discussion

In this study, we investigated in further detail the delayed use of EBOTAb, a cost-effective antibody treatment for EBOV. Whilst responses when EBOTAb has been delivered 6, 48 and 72 hours post-challenge have previously been reported^20^, in this study dosing was started at either 3, 4 or 5 days post-challenge. Our results show that prior to initiating treatment, viral RNA was present in the bloodstream 3 days post-challenge, before rising subsequently after 4 and 5 days. This is consistent with other guinea pig studies, where viremia was first detected within 2 days post-challenge and increased to a peak on day 7^25^. In this study we assessed viral load with the standard diagnostic PCR assay for EBOV^26^; thus, our study followed the same methodology used in human EBOV infection to ensure that the guinea pigs were first given EBOTAb in the same treatment window used in the clinical setting.

Delayed treatment with a cocktail of 3 pooled mAbs has demonstrated protection of 67% (4/6 guinea pigs surviving) when given 3 days post-challenge^27^; similar to our results with EBOTAb which demonstrated 83% efficacy (5/6 guinea pigs surviving) when treatment was also first administered day 3 post-challenge in the current study and 75% efficact (3/4 guinea pigs surviving) in a previous study^20^. In the pooled mAb study, treatment was initiated at -1, 1, 2 and 3 days post-challenge and demonstrated that survival peaked with delivery starting at day 2 post-challenge with 100% protection, but when delivered 1 day prior or 1 day after challenge the efficacy dropped to 50%^27^. Our results are more in line with expectations of efficacy waning with increasing time to initiating treatment, with efficacies of 50% and 33.3% observed when EBOTAb was first delivered at days 4 and 5 post-challenge, respectively.

In our study, we compared the effects of EBOTAb with ZMapp, one of the leading mAb therapies against EBOV which contains a mixture of 3 mAb produced from the plant, *Nicotiana benthamiana*^28^. Our results demonstrate that when EBOTAb and ZMapp are first administered 3 days post-challenge, there is no statistical difference in survival outcomes if the EBOTAb is then administered throughout the course of infection. When EBOTAb was given only on day 3 post-challenge, it still provided a statistically significant level of protection although this was not as effective as repeat dosing of EBOTAb or with ZMapp delivered on a single day. Interestingly, despite their being no significant difference in survival at day 3 of EBOV infected guinea pigs between those treated with 12 doses of EBOTAb or with a single dose of ZMapp, the clinical condition of animals which received ZMapp was superior. Thus, animals treated with ZMapp showed no loss in weight, no temperature fluctuations nor clinical signs throughout the period of study, unlike those that received EBOTAb. Whilst survival is the most important aspect of an EBOV therapy, these results also indicate that treatment with EBOTAb might require supportive care in addition to direct symptom management associated with disease.

The use of a pAb therapy negates limitations associated with mAb-based therapies, particularly the risk that mutations can arise in epitopes that could render escape from therapeutic regime and necessitates the development of multiple antibody cocktails^29^. From several studies of mAb treatment using a combined total of 36 NHPs, the occurrence of escape mutations was suggested to be 3%. While this was only detected in a single animal^30^, their development is significant. Additional evidence was produced in a study using cynomolgus macaques treated with a combination of three mAbs (ZMAb) where mutations in the epitope binding sites of these antibodies were detected^31^, suggesting the level of escape mutations to be higher. Similar mutations have also been identified in rhesus macaque studies using MB-003, a similar mAb combination therapy to ZMAb and ZMapp^22^. Given that NHP studies are controlled and conducted within a relatively short timescale, it could be speculated that escape mutations following treatment with mAb-based therapeutics in human EBOV infection could potentially compromise the ability to contain outbreaks in affected populations. This might be exacerbated by the ability of EBOV to lie dormant within immune privileged sites after clinical disease^32^,^33^.

As filoviruses are endemic in developing and third-world countries, it is important that therapeutic costs are kept to a minimum so that stockpiling of treatments for emergency purposes is not an economic burden^14^. It has been suggested that existing contract manufacturing organisations under ideal conditions could produce mAbs at an unit cost as low as US$60/g^34^. However, the costs incurred to develop mAbs into approved treatments are high. For example the US Biomedical Advanced Research and Development Authority (BARDA) provided financial support of US$18.9 m over 18 months to Mapp Biopharmaceutical to generate a relatively small stock of clinical grade (cGMP) ZMapp (<1000 doses) to advance the product through clinical trial evaluation^35^. Additionally the production of mAb from tobacco plants is not a straightforward process^36^, a cheaper approach for the production of mAb is to use mammalian expression systems. However, it has also been shown that when one of the ZMapp precursor mAb combinations (MB-003) was expressed in a mammalian expression system there was a threefold difference in effectiveness when tested in rhesus macaques compared to the same mAb cocktail being expressed with tobacco virus^28^. This observation has also been reported in mice^37^. The only change between ZMapp produced in tobacco plants and in Chinese hamster ovary (CHO) cells is the glycosylation of the antibodies. The absence of core fucose in the ZMapp antibodies is known to increase binding to the FcγRIII, resulting in a dramatic improvement in antibody-dependent cellular cytotoxicity (ADCC)^28^. As mammalian cells in general produce only a small percentage of antibodies lacking core fucose, it might be for this reason that ZMapp performs better than EBOTAb in reducing viremia and clinical signs of EBOV in the guinea pig studies we report within.

EBOTAb and the ZMapp-precursor mAb combinations have both previously been tested at 3 days post-infection in EBOV-infected guinea pigs, demonstrating 75%^20^ and 33–67%^18^,^38^ efficacies, respectively. To the best of our knowledge there is currently no data where antibodies against EBOV have been administered after day 3 post-challenge in the guinea pig model. The finding that EBOTAb exerts a level of protection as late as day 5 post-challenge is therefore a significant finding. A major difference between the effects of EBOTAb and ZMapp was on viral load at day 8 post-challenge; a timepoint chosen since circulating viral loads are high^8^ and, historically, this is when guinea pigs begin to meet humane clinical endpoints. Whilst there was no difference in circulating viral RNA levels in the EBOTAb treated animals, no EBOV RNA was detected in those which received ZMapp. Therefore, EBOTAb might have different mechanisms of action. Both EBOTAb and ZMapp are known to have high neutralisation activity against live EBOV^11^,^16^; however, in order to directly compare, further work would be required to test both compounds in identical assay systems. Since EBOTAb is a pAb preparation it binds to multiple epitopes on the EBOV glycoprotein which play different roles in the course of viral infection^24^.

The finding that EBOV RNA was present in liver and spleen (the only organs sampled and tested) indicates residual virus present at the end of the study, including in the animals treated with ZMapp. The range of responses observed is typical due to the use of outbred guinea pigs. However, given that no EBOV antigen was detected by immunostaining in several of the samples showing positive PCR, it is likely that the PCR assay detected viral nucleic acid fragments remaining from the infection. A similar finding has been reported by others who showed detection of viral RNA at 28 days post-challenge but live virus was not recovered^39^. Interestingly, no viremia was detectable in the blood of animals tested at 21 days post-challenge, the scheduled end of the study. This is important, as negative results of PCR assays in the blood are currently one of the main parameters for the discharge of EBOV convalescent patients^40^.

In our study, we have evaluated the antibody preparations as a sole treatment option. It may be possible that the protective efficacy of EBOTAb could be increased by supplementing with other compounds. For example: A human adenovirus serotype 5 which expresses recombinant interferon-α (IFN-α), termed DEF201, has been shown to enhance protection of an adenovirus-based vaccine against EBOV when delivered 30 minutes post-exposure in guinea pigs^41^. Rapid administration of DEF201 has also been shown to result in complete survival of EBOV infected guinea pigs when mAbs were delivered within 7 days post-infection^17^. A similar result was observed with DEF201 and mAbs in NHPs^42^. The DEF201 could constitutively produce high levels of IFN-α for days in a cost efficient manner, as recombinant IFN-α is expensive and has a short half-life of 8 hours *in vivo*^43^.

Whilst an uninfected EBOTAb treated group was not conducted, it is unlikely that any adverse effects would be detected. Our previous studies have demonstrated that when treatment is first given either 6 or 48 hours post-challenge with EBOV, no clinical signs were detected when EBOTAb was delivered on 10 or 8 occasions, respectively^20^. A similar based therapy against *Clostridium difficle* has also been developed using the same platform^44^ and has completed GLP toxicity studies in rats with no adverse findings reported. Whilst a case of severe anaphylactic/anaphylactoid reaction has been reported to an ovine polyvalent immune preparation after a snakebite injury^45^, this was later challenged by other authors with the clinical findings to be more consistent with angioedema caused by angiotensin converting enzyme (ACE) inhibitors^46^. Therefore, this example of a serious adverse reaction to an ovine antibody-based preparation was compromised by the patient an anti-hypertensive medicaction with a design based on venom, was envenomated, and was then treated with another venom-derived medication^45^. Additionally, our results demonstrated an antibody response being raised against EBOTAb. However, despite the small numbers of animals studied, there was no evidence of titres increasing with repeated deliveries of EBOTAb.

In this work, we have used the guinea pig model as a suitable small animal model for EBOV disease. While murine models exist, these are less predictive to study efficacy of treatments and vaccines compared to guinea pigs and non-human primates (NHPs)^38^,^47^,^48^. The guinea pig model displays the same tissue tropism as in NHPs and recapitulates the lesions observed in tissues of EBOV infected NHPs and humans^25^. The histological findings of diffuse lymphocyte depletion, necrosis and congestion in the spleen and liver samples of EBOV infected guinea pigs in this report are also features observed in EBOV infected NHPs^49^,^50^. Nevertheless, future studies are underway to analyse the efficacy of EBOTAb in the NHP model.

Whilst a post-exposure treatment would confer significant public health benefits to communities affected by EBOV, a medical countermeasure would also be of use in the event of laboratory exposure to the virus as has previously occurred, including institutions in the US^51^, Côte d’Ivoire^52^, England^53^ and Germany^54^. The recent outbreak in West Africa has also highlighted the nosocomial transmission of this virus to healthcare workers^55^. In addition, EBOV has historically, and in the present day, been listed as a possible agent for bioterrorism^56^.

Our results demonstrate the potential importance of EBOTAb as a cost-effective therapy against EBOV and further strengthen the evidence base showing protective effects as late as 5 days post-challenge. The advantages of the pAb technique in reducing the risk of escape virus mutants further strengthens the continued development of EBOTAb as a potential therapy for EBOV. In addition, EBOTAb was developed rapidly in response to the EBOV outbreak in a fraction of the time that has been spent developing alternatives, which demonstrates the feasibility of the ovine pAb approach to other emerging pathogens that may arise in the future.

## Methods

### Ethics statement

Animal studies were performed under Containment Level 4 conditions with all procedures being undertaken according the United Kingdom Animals (Scientific Procedures) Act 1986. Studies were conducted under Establishment Licence reference PEL PCD 70/1707 with Project Licence PPL 30/3247. Studies were approved by the Public Health England ethics committee and the Project Licence approved by a UK Home Office inspector.

### Virus and antibody preparations

EBOV strain Yambuku-Ecran (previously named ME718^57^) was passaged five times in guinea pigs to achieve lethality, as previously described^58^. Virus was titrated by 50% tissue culture infective dose (TCID_50_) assay in VeroE6 cells (European Collection of Cell Cultures, UK). EBOTAb was generated as previously reported^20^. ZMapp was supplied by Mapp Biopharmaceutical, Inc. Where required, EBOV and antibodies were diluted with sterile PBS.

### Animal experiments

Female adult Dunkin-Hartley guinea pigs (Harlan Laboratories, United Kingdom) were used for *in vivo* studies, with an average mean starting weight of 325 g (range 288–359 g). Animals were supplied with catheters inserted into the jugular vein to allow intravenous access^59^. For procedures, guinea pigs were anaesthetised with 1.5%–2% isoflurane in oxygen until full sedation was achieved. Food and sterile water were available *ad libitum*. Animals were weighed and temperatures recorded daily via an indwelling temperature chip. Clinical signs were monitored at least twice daily, and the following numerical score was assigned for analysis: 0 (normal); 2 (ruffled fur); 3 (lethargy, pinched, hunched and wasp waisted); 5 (labored breathing). EBOV stock was diluted in sterile phosphate buffered saline (PBS) to prepare 10^2^ TCID_50_ in a 0.2 mL volume and subcutaneously inoculated in the lower right quadrant of the back of each animal. Virus was back titrated on VeroE6 cells to confirm the dose. EBOTAb was delivered intravenously starting at either 3, 4 or 5 days following EBOV challenge. Repeat administrations (2 doses) were delivered on each of the following two days and then on alternate days for another 3 occasions. On each day of administration, twice-daily dosing of 0.5 mL EBOTAb (51 mg/mL) was delivered with at least 5 hours between doses. One group of animals received EBOTAb on day 3 post-challenge only. EBOTAb was diluted to 48 mg/mL and 0.5 mL was delivered over three occasions separated by at least 3 hours. ZMapp was diluted to 3.4 mg/mL, and administered identically to the animals that received EBOTAb on day 3 only. For the animals which received compounds only on day 3 post-challenge, the amounts received were 72 mg/animal EBOTAb (5 mg/mL EBOV-specific antibody) and 5 mg/animal ZMapp.

### EBOV titration by RT-PCR

Blood samples were taken in RNAprotect animal blood tubes (Qiagen, UK), mixed and stored at −80 °C until processing. For processing, 200 μL of tissue homogenate or blood solution was transferred to 600 μL RLT buffer (Qiagen) for RNA extraction and PCR. Extraction of RNA was performed using the MagnaPure 96 small volume RNA kit (Roche). Plates were loaded onto the MagnaPure 96 automated extraction robot and RNA was eluted in 60 μL nuclease free water. Target amplification was performed using primers to EBOV glycoprotein^60^ using the Fast Virus qRT-PCR Kit (Qiagen). Analysis was performed using the ABi 7500 (Applied Biosystems) at the following cycling conditions; 50 °C for 10 minutes, 95 °C for 30 seconds followed by 40 cycles of 95 °C for 15 seconds and 60 °C for 3 seconds. Temperature cycling was set to maximum ramp speed and data was acquired and analysed using the ABi 7500 on-board software with a threshold set to 0.05. A standard curve of RNA transcript at known quantities was run in parallel on each PCR plate for assessing genome copies per reaction.

### Histology

Samples of liver and spleen were placed in 10% neutral buffered formalin for at least 21 days and processed routinely to paraffin wax. Sections were cut at 3–5 μm, stained with haematoxylin and eosin (HE) and examined microscopically. For immunohistochemistry, sections were stained for EBOV antigen using the Leica BondMax (Leica Biosystems) and the Leica Bond Polymer Refine Detection kit (Leica Biosystems). An antigen retrieval step was included for 10 minutes using the Bond Enzyme Pretreatment kit, enzyme 3 (3 drops). A rabbit polyclonal, anti-EBOV VP40 antibody (IBT Bioservices) (dilution 1:2000) was incubated with the slides for 60 minutes. DAB chromogen and haematoxylin counterstains were used to visualise the slides.

### Anti-EBOTAb antibody ELISA

High binding ELISA plates (Maxisorb, Nunc) were coated with 10 μg/ml EBOTAb diluted in PBS overnight at 4 °C. Unbound antibody was removed by washing three times with PBS and a three-fold dilution of sera was conducted starting at 1:10 and added to the plate. All conditions were tested in triplicate wells. After 1 hour at 37 °C plates were washed three times with PBS and a peroxidase-labelled donkey anti-guinea pig IgG antibody solution added (Jackson ImmunoResearch). After 1 hour at 37 °C plates were washed 5 times with PBS and 2,2’-Azinobis [3-ethylbenzothiazoline-6-sulfonic acid]-diammonium salt (ABTS) substrate (Insight Biotechnology) added and left for 15 minutes at room temperature before the reaction was stopped by the addition of ABTS stop solution (Insight Biotechnology). Absorbances were read on a plate spectrophotometer at a wavelength of 405 nm.

### Statistics

To determine group sizes for the *in vivo* studies, a power calculation with the Fisher exact test was performed using software G*Power, version 3.0.10. A group size of 6 met a power of 0.8 and an α of 0.05. The Log-Rank test for nonparametric survival was used to compare differences between groups of animals using Minitab, version 16 and applying right-censoring with a time censor of 21 days.

## Additional Information

**How to cite this article**: Dowall, S. D. *et al*. Post-exposure treatment of Ebola virus disease in guinea pigs using EBOTAb, an ovine antibody-based therapeutic. *Sci. Rep.* **6**, 30497; doi: 10.1038/srep30497 (2016).

## Figures and Tables

**Table 1:** Histological changes and immunohistochemistry staining in the liver and spleen of EBOV challenged guinea pigs treated with EBOTAb and ZMapp.

	Animal ID (day*)	Liver				Spleen					
		Lipid vacuoles	Focal necrosis	Focal mineral	IHC	Congestion	Red pulp single cell death	Red pulp foci PMN	White pulp single cell death	White pulp lymphocyte depletion	IHC
Untreated	03893 (7)	−	+	−	+++	+++	+++	++	+++	+++	+++
	03915 (7)	++++	++	−	++	++	++	+++	+	+	+++
	03917 (7)	+++	+	+	++	++++	++++	+++	++	+++	++
	89256 (7)	+++	++	+	+++	+++	++++	+++	+++	+++	+++
	89299 (8)	++	+	−	++	+++	++	−	+++	++++	++
	03904 (11)	++++	++	+++	++	+++	++	+++	+	+	+
EBOTAb starting 3 days post−challenge**	89314 (8)	+	++++	+++	++++	+++	++++	−	++++	++++	++++
	89273 (21)	−	−	++	−	+	−	−	−	−	+
	89209 (21)	+	−	−	−	−	−	−	−	−	+
	89303 (21)	−	−	++	+	−	+	+	+++	++	+
	89258 (21)	−	++	−	+***	−	−	−	−	++	+
	89265 (21)	−	−	−	−	−	−	−	−	−	−
EBOTAb starting 4 days post−challenge**	89271 (9)	+++	++++	++++	++++	+++	++++	++	++++	++	++++
	89318 (12)	+++	++++	+++	++	−	++++	−	++++	+++	+++
	89311 (13)	+	+++	++++	+++	++	+++	++++	+	+++	+
	89278 (21)	+	+	++++	+	−	−	−	−	−	+
	89349 (21)	−	−	++++	+	++	++	−	+	++	+
	89336 (21)	−	++	−	−	−	−	−	−	−	+
EBOTAb starting 5 days post-challenge**	89253 (6)	+++	++++	−	+++	−	++	+++	+	++	++
	03892 (7)	+++	++++	+	++	++	++++	+	−	−	++
	89344 (9)	+	++++	++++	+++	++++	+++	+	+++	+++	+++
	89352 (12)	+++	++++	+++	+++	+++	++++	+++	++	++	+
	89245 (21)	−	−	++++	+	−	−	−	++	−	+
	89257 (21)	−	−	−	+	−	−	−	−	−	−
EBOTAb, day 3 only	03911 (8)	+	+	+	−	+++	++	−	−	+	−
	89279 (9)	+++	+++	++	++	+++	++++	+++	++++	++	++
	89282 (9)	−	++++	−	+++	++++	++	−	+++	++	+++
	03898 (12)	++++	+	++++	+	+++	++++	+++	+++	++++	+
	03883 (21)	−	−	+	−	−	−	−	−	−	−
	89312 (21)	−	−	++++	+	−	−	−	−	−	+
ZMApp, day 3 only	03905 (21)	+++	−	+	−	−	−	−	−	−	−
	03886 (21)	+	−	−	−	+	−	−	−	−	−
	03907 (21)	−	−	−	−	−	−	−	++	−	−
	03971 (21)	+++	−	−	−	−	−	−	−	−	−
	03913 (21)	+	−	−	−	−	−	−	−	++	−
	03887 (21)	+	−	−	−	−	−	−	−	−	−

Abbreviations:-within normal limits; +minimal; ++mild; +++moderate; ++++marked.

*day post-challenge samples were collected; **repeat dosing of EBOTAb was conducted on 5 subsequent days; ***unusual intra-nuclear immunostaining.
